# Five mucosal transcripts of interest in ulcerative colitis identified by quantitative real-time PCR: a prospective study

**DOI:** 10.1186/1471-230X-8-34

**Published:** 2008-08-12

**Authors:** Anders Eriksson, Carl-Fredrik Flach, Anders Lindgren, Eva Kvifors, Stefan Lange

**Affiliations:** 1 Department of Internal Medicine, Gastroenterology Unit, Sahlgren's University Hospital/Östra, Göteborg, Sweden; 2 Institute of Biomedicine, Department of Microbiology and Immunology, Göteborg University, Gothenburg, Sweden; 3 Department of Internal Medicine, Borås Lasarett, Borås, Sweden; 4 Institute of Biomedicine, Department of Clinical Bacteriology, Göteborg University, Gothenburg, Sweden

## Abstract

**Background:**

The cause and pathophysiology of ulcerative colitis are both mainly unknown. We have previously used whole-genome microarray technique on biopsies obtained from patients with ulcerative colitis to identifiy 5 changed mucosal transcripts. The aim of this study was to compare mucosal expressions of these five transcripts in ulcerative colitis patients vs. controls, along with the transcript expression in relation to the clinical ulcerative colitis status.

**Methods:**

Colonic mucosal specimens from rectum and caecum were taken at ambulatory colonoscopy from ulcerative colitis patients (*n *= 49) with defined inflammatory activity and disease extension, and from controls (*n *= 67) without inflammatory bowel disease. The five mucosal transcripts aldolase B, elafin, MST-1, simNIPhom and SLC6A14 were analyzed using quantitative real-time PCR.

**Results:**

Significant transcript differences in the rectal mucosa for all five transcripts were demonstrated in ulcerative colitis patients compared to controls. The grade of transcript expression was related to the clinical disease activity.

**Conclusion:**

The five gene transcripts were changed in patients with ulcerative colitis, and were related to the disease activity. The known biological function of some of the transcripts may contribute to the inflammatory features and indicate a possible role of microbes in ulcerative colitis. The findings may also contribute to our pathophysiological understanding of ulcerative colitis.

## Background

Ulcerative colitis (UC) is a disorder characterized by chronic mucosal inflammation of the large intestine. It is frequently associated with various extraintestinal manifestations. The inflammation may be limited to the rectum (proctitis), but mucosal lesions often continue more proximally (left-sided UC) or additionally embrace the transverse colon (extensive colitis) or the entire large bowel (pancolitis). The immune and cellular (non-immune) response is dysregulated in both the acute and the chronic phase of UC [1,2]. In Scandinavia, UC has been found to affect individuals of all ages, with an annual incidence of about 15 per 100 000 [3,4] and a prevalence of about 300 per 100 000 inhabitants [5].

The pathogenesis and pathophysiology of UC are still under investigation [6]. We can tentatively say that the cause and onset of the disease is polygenic with environmental interaction; that is, there is a genetic predisposition [7-9] in combination with eliciting environmental factors which may precipitate the phenotype of UC [10]. In addition, interaction between the colonic epithelium and microbiological flora as well as a disintegrated mucosal barrier function may be important factors in the onset and development of UC [6]. The use of microarray technique analyses on mucosal specimens obtained from both patients with established UC and controls has allowed identification of candidate genes, which are valuable in research on UC pathogenesis. However, these UC candidate genes must be carefully selected, since recent evaluations of microarray data have revealed considerable divergence after examination of similar tissues [11-13]. Such divergent results are commonly presented in studies using pooled patient samples. In the present study however, the transcripts selected are based on our earlier individual whole-genome microarray screening and quantitative real-time PCR (RT-PCR) in patients with UC [14], where five changed genes/transcripts were identified; aldolase B, elafin, MST-1, simNIPhom (similar to NIP homolog), and SLC6A14. The pathophysiological properties of SimNIPhom have not yet been clarified, but the other transcript products have potential importance in secretion [15,16], anti-microbiological activity [17], and cell-mediated immune response [18].

The primary aim of the present study was to define differences in the mucosal expression of five selected transcripts, retrieved from two different colonic locations in UC, by using a quantitative RT-PCR technique. We also aimed to evaluate the influence of ongoing anti-inflammatory treatment as well as the importance of the colonic UC extension and the severity class.

## Methods

### Patients and tissue specimens

Before the colonoscopy procedure, consecutive male and female subjects (UC patients and controls, >18 y) were recruited to the present study. The UC diagnosis was based on the medical history, endoscopic findings, histological examination, laboratory tests, and the clinical disease presentation. The extent of UC and the clinical activity were classified in accordance with the Montreal Classification [19]. In brief, the colonic inflammatory involvement is defined as extension (letter E) combined with a number between 1–3 (E1 denotes proctitis, E2 left-sided UC, and E3 extensive colitis). In addition, the clinical severity grade (letter S) is defined. The S-score ranges from clinical remission (S0) to severe UC (S3). Mucosal biopsies were obtained from the rectum (10–15 cm proximal from anal verge) and caecum from all participants.

No uses of corticosteroids, aminosalicylates, or immunosuppressants were registered in the control group, while 9 patients (18%) in the UC group were treated systemically with corticosteroids (prednisolone 10–20 mg). Twenty-seven UC patients (55%) were treated systemically with aminosalicylates (mesalazine 1.600–2.400 mg/24 h). Among these, two patients had additional ongoing therapy with aminosalicylate (mesalazine 500 mg QD) enemas and three patients were treated with corticosteroid enemas (prednisolone 37.5 mg QD or BID). Seven patients had stable (>3 months) ongoing immunosuppressant treatment (Azathioprine, 1.8–2.2 mg/kg bw).

**Figure 1:**
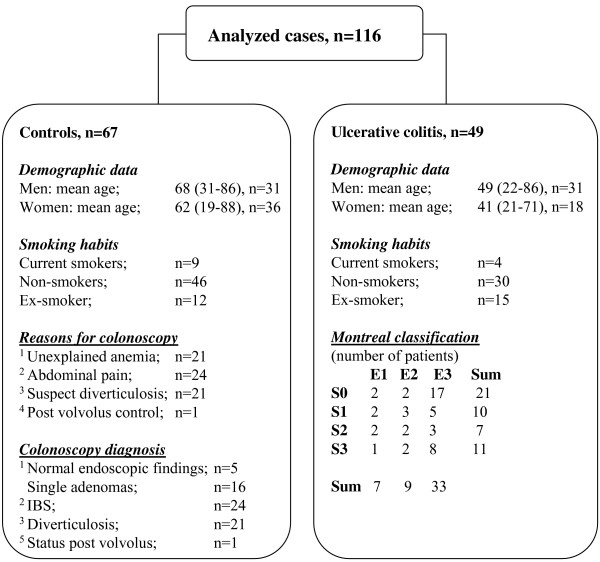
Demographic and clinical data from the control and UC group respectively.

The remaining demographic and clinical data are presented in Figure 1.

### RNA isolation

The biopsy specimens were immediately stored in RNA-later solution for isolation of RNA. The RNA-later-preserved biopsies were homogenized in a lysis buffer from the GenElute Mammalian Total RNA kit (Sigma, St. Louis, MO.) and total RNA was isolated according to the manufacturer's instructions. The RNA concentration was measured spectrophotometrically.

### Quantification by real-time polymerase chain reaction (RT-PCR)

Two μg of total RNA from each sample were converted into cDNA. The cDNA synthesis was performed as described previously [20]. Oligonucleotide primers purchased from MWG-BIOTECH AG (Ebersberg, Germany) were used for the relative quantification (ABI-7500 system, software version 1.3) (Table 1). Glyceraldehyde-3-phosphate dehydrogenase (GAPDH) was used as a reference gene in all experiments. The expression level in each sample was compared with a calibrator by using the ΔΔC_T- _formula (ΔC_T(calibrator) _- ΔC_T(sample)_).

**Table 1:** Primers used for real-time polymerase chain reaction.

**Gene**	**Forward primer**	**Reverse primer**
**Aldolase B**	5'-aaggctgcaaacaaggaggcaacc-3'	5'-tgaagagcgactgggtggaagcag-3'
**Elafin**	5'-tgtgaaggctcttgcgggatgg-3'	5'-agggcagcagggacttaggaccag-3'
**SimNIPhom**	5'-cgccagacagctaggggagtgaag-3'	5'-gcatttctgatattttgtgaccacgcac-3'
**SLC6A14**	5'-gctgcttggttttgtttctccttggtc-3'	5'-gcaattaaaatgccccatccagcac-3'
**MST-1**	5'-aaccaggagtgtaacatcaagcaccgag-3'	5'-cagttgtgggtaaagcaggcaagtgg-3'
**GAPDH**	5'-gagcaccaggtggtctcctctgacttc-3'	5'-gccaaattcgttgtcataccaggaaatg-3'

### Statistical analysis

Descriptive statistics and the Wilcoxon signed rank test (SAS, Statview^®^) were used. Median values are presented.

### Ethics

The study was approved by the local research ethical committee. All patients were given oral and written information before entering the study. Informed consent was obtained from all patients and controls.

## Results

The mean duration of UC was 9.3 years (proctitis 9.6 years, left-sided colitis 9.5 years and extensive colitis/pancolitis 9.2 years). Neither age nor gender was matched between the UC group and the control group.

In order to evaluate any differences in transcript expressions within the control group (n = 67) with respect to background diagnoses (anaemia, diverticulosis, irritable bowel disease and polyposis), statistical analysis of each background diagnosis were compared to the remaining group of controls. No significant differences (p > 0.05) were detected for any of these diagnoses.

**Figure 2:**
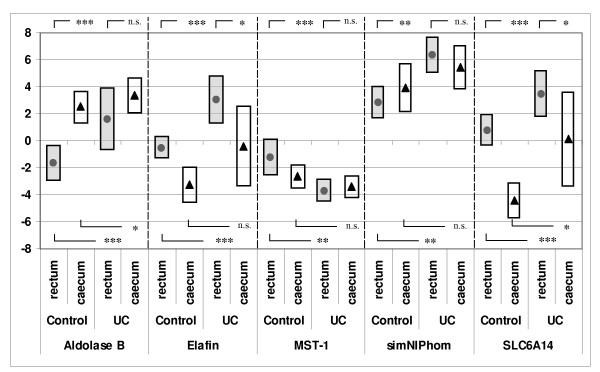
**RT-PCR result (ΔΔCt(=ΔCt_target_-ΔCt_calibrator_)) for controls (filled dots) and UC patients (▲) presented as median values and 25^th ^and 75^th ^percentil (bars).** * = *p *< 0.05, ** = *p *< 0.01, *** = *p *< 0.001, n.s. = non significant. Reference gene: GAPDH.

Significantly higher transcript expressions of aldolase B and SimNIPhom and significantly lower transcript expressions of elafin, MST-1, and SLC6A14 were found in caecal biopsies in comparison to rectal biopsies from the control group (Figure 2). The only significant differences between rectal and caecal transcript expressions in UC patients were the decreased transcript expressions of elafin and SLC6A14 in caecal biopsies in comparison to rectal biopsies.

Comparison of rectal biopsies from controls (n = 67) with rectal biopsies from UC patients with inflammatory activity in accordance with Montreal classifications S1–S3 (n = 28) showed significant elevations (p < 0.05) in UC patients of all transcript expressions with the exception of MST-1, which showed significantly (p < 0.05) decreased expression in UC patients. The same analysis of caecal biopsies from controls and patients with S1–S3 UC (n = 16) showed significantly elevated transcript expressions of aldolase B and SLC6A14 only. Distal biopsies from controls, compared with UC patients without inflammatory activity (S0), showed increased transcript expression in aldolase B only (median -1.62 vs. 1.0, p = 0,012). All other transcript analyses from both locations showed no significant differences (p > 0.05).

All transcript analysis with respect to UC extension showed that left-sided (E2) and total colitis (E3) differed significantly from controls (p < 0.05); this was not the case for proctitis (E1).

Statistical analysis concerning the influence of anti-inflammatory treatment on the transcript expressions within the UC cohort showed no statistical differences (p > 0.05) when comparing UC patients with ongoing corticosteroids (n = 9) or azathioprine (n = 7) respectively with the remaining UC patients. However, the 27 patients treated with mesalazine show a significant increase in aldolase B (median 0.48 vs. 3.02, p = 0.035) in comparison to the remaining UC patients.

## Discussion

Genetic predisposition, psychological stress, nutritional and environmental influences, intestinal pathogens and disturbed intestinal barrier function have all been proposeas pathogenetic factors in UC [6]. However, current knowledge about the pathogenesis and pathophysiology of UC [21] is incomplete. Moreover, with the exception of a few general serological inflammatory activity biomarkers, even less information is available regarding mucosa-associated transcript changes and their potential pathogenetic and pathophysiological role in UC [22]. This lack of knowledge may sometimes lead to uncertainty in diagnosis, judgement of prognosis and clinical management of UC patients.

On the basis of exsisting knowledge of the biological functions of the transcripts investigated in this study, it is reasonable to believe that the demonstrated alterations might be related to predisposition and/or the pathophysiological response in UC.

It is intriguing that aldolase B and SLC6A14 were up-regulated in rectal as well as caecal mucosa in UC compared to controls. Aldolase B is known to be mainly expressed in the intestinal villus cells and it has a central role in the glycolytic pathway. It also participates in regulation of intestinal secretion [16]. Since SLC6A14 is also known to encode a Na^+^/Cl^- ^driven amino acid transporter B(0+) [15], the up-regulation of aldolase B and SLC6A14 might be a common pathophysiological response, aimed at counteracting the exaggerated loss of fluid seen in UC. Theoretically, the up-regulation of these two transcripts could be a local response to the increased feacal/fluid stream, where bioactive molecules comprise ability to regulate transcript expression. Additionally, since the inflammatory activity and load of fluid over time is usually most pronounced in the distal part of the colon, the registered changes in aldolase B and SLC6A14 may reflect long-term inflammatory activity. Our finding that aldolase B from distal biopsies is significantly elevated during the remission phase (S0) indicates that the regulation of this transcript not only is secondary to the inflammatory activity.

The involvement of the microflora and its importance in the onset, development and preservation of UC has been discussed [6]. The SLC6A14 transcript expression is therefore also interesting in this respect, since it is involved in the host's antibacterial response [21]. In addition, the defensine-like epithelium associated antimicrobial molecule elafin, antagonizing human neutrophil elastase preventing tissue injury via inhibition of excessive release of proteolytic enzymes from inflammatory cells is interesting in this context [18]. The present results confirm an elafin transcript enhancement in caecal as well as rectal biopsies from patients with UC. Thus, the combined elevation of elafin and SLC6A14 may contribute to an amplified defence reaction aimed to restoration and maintenance of the mucosal integrity. This finding may indicate a pathogenetic role of the microflora in UC.

MST-1 was included in the present study due to its alterations in UC, as shown in our previous experiment [14], although it was excluded from that publication due to deviation in its control group. MST-1 is known to be capable of inhibiting cell-mediated immune responses via down-regulation of IL-12 production and subsequently inhibition of macrophage activation [23]. Consequently, the observed down-regulation of MST-1 in rectal specimens may contribute to an enhanced cellular immune response in UC. A reasonable explanation of the concomitant decreased MST-1 transcript expression and increased aldolase B, SLC6A14, and elafin transcript expression is that the changes describe a pathophysiological response to a more pronounced inflammatory and, possibly, an exaggerated microbial load in at least the rectal part of the colon mucosa.

The fifth identified significantly up-regulated transcript (in rectum only) SimNIPhom (similar to the numb-interacting homolog), encodes a hypothetical protein, at present of unknown pathophysiological importance.

Our results supports that specimens from the rectal mucosa are more suitable for further analysis of the selected transcripts, due to the more predictable inflammatory involvement in the rectum and its availability for direct inspection and easy biopsy sampling.

Our data can not answer whether the observed changes in expressions of the five selected transcripts may be present in e.g. other inflammatory, infectious or autoimmune conditions since this study uniquely focused on UC patients compared to non-inflamed controls.

## Conclusion

The five changed gene transcript expressions have relation to UC, its extension and clinical severity. Whether the presented results will contain discriminative potential of importance for the medical care of patients with UC in future clinical practice remains to be elucidated.

## Competing interests

The authors declare that they have no competing interests.

## Authors' contributions

AE designed the study, preformed sampling of biopsies, analyzed the data, and prepared the manuscript. C-FF analyzed the data. AL preformed sampling of biopsies. EK coordinated the study. SL designed the study, analyzed the data and prepared the manuscript. All authors read and approved final manuscript.

## Pre-publication history

The pre-publication history for this paper can be accessed here:


